# Letter from Dr. Harlan

**Published:** 1839-08-03

**Authors:** 


### FOREIGN CORRESPONDENCE.

### LETTER FROM DR. HARLAN.—No. V.

Paris, *June*, 1839.

Of the numerous operations resulting from the gunshot wounds during the "emeute," or insurrection of the 12th and 13th of May, a great many of the more grave cases have terminated fatally. The operation for disarticulation at the shoulder, is, I am informed, usually unsuccessful; inflammation of the veins, and suppuration caused so near to the heart, appear to be the prominent cause of this fatality. The patients that I visited, under these circumstances, appeared to do well at first, but were suddenly affected with chills in a few days, and sunk.

In my last communication I alluded to the different operations of Velpeau and Breschet for varicose veins. Recently, M. Ricord has resorted to another method, which he esteems superior to either, viz., he pinches up the skin, including the diseased vein, and passes a needle, armed with a ligature, *beneath* the vein; he then passes the needle back again through the same holes *above* the vein, draws the knot, and thus obliterates the vein; and, in a few days, the ligature comes away. His cases, thus far, have ended successfully.

M. Brocchieri, of Naples, a grandiloquent charlatan, for some time resident in Paris, has recently made some noise in the medical circles here. He complains of the want of cordiality of the French physicians, notwithstanding the apparent candour of his statements, and liberal elucidations by experiments on living animals. M. B. professes to have discovered a chemical fluid capable of arresting the hæmorrhage immediately and permanently, arising from wounds of even the largest arteries; he has christened this fluid the "Balsamelœon, eau Hémostatique et Antiscorbutique de M. Pierre Brocchieri, Napolitain." He holds this fluid as far surpassing in power the far-famed *Aqua Binelli*, a hemostatic remedy, the secret of which died with its author.

On the 29th of May last, M. B. invited, by a printed circular, all the prominent members of the profession, native and foreign, to witness a suite of experiments upon living animals, which should test the undoubted value of his discovery. At the appointed hour I answered, with numerous others, what he was pleased to call an "Appel à la Science Médicale au nom de l'humanité."

The carotid artery of a sheep was laid bare, and opened obliquely. Large portions of lint, previously soaked in his eau hémostatique, were applied to the bleeding vessel, and pressed by the hand for fifteen minutes, more of the water being occasionally poured on the lint thus applied; a roller bandage was then applied, to confine the lint over the artery for one hour and a half, when all the dressings were removed, rather roughly, as the lint adhered pretty firmly to the wound; the hæmorrhage was entirely suppressed. We witnessed similar experiments repeated on different vessels of various sizes, on different animals, and with similar results. In one instance, the tail of a large dog was amputated with a sharp instrument, and the bleeding was easily suppressed.

M. Amusat, and some other French surgeons, pretended to be still sceptical, and thought that it was possible that similar results might possibly have followed a similar application of simple cold water; and, a few days after, this notion was tested by Lisfranc, at the Hôpital La Pitie, but the experiments resulted in favour of M. Brocchieri.

The "Eau Hémostatique" is colourless, spicy to the taste, and of a somewhat tarry odour. It is administered internally, also, by M. B., who states that it is not only innocent, but capable of curing all the worst diseases which afflict humanity, and by this over-praise he has averted the serious attention of many physicians. I could discover not the least astringency in the taste of this liquid, which seems really to form a chemical union with the fibrine of the blood, causing a firm coagulum, which closes the wounds made in the arteries.

M. B. made large incisions in the flesh of animals, when the cavity, as usual, was immediately filled with blood; but the oozing of blood was immediately suppressed by sponging with the fluid. Dr. Mott informed me that he had previously assisted at similar experiments, and that in one instance he cut the carotid artery of a sheep entirely across.

M. Brocchieri is the same person who published documents on this subject several years ago in Naples; and experiments similar to the above were witnessed at that time by Dr. Barabino, of Philadelphia, and several other American naval surgeons.

I send you some printed documents of M. B., which will enable you to form some idea of this singular author.

As a subject of less "questionable shape," I must now allude to a noble institution, founded on the philosophical application of the laws of physics to anatomy and physiology,—I speak of the "Institution Orthopédique" of Dr. Guérin. This delightful mansion is situate near Passy, in the Bois de Boulogne; it is known as the "Château de la Muette," which formerly belonged to the crown, but is now private property. By far the most interesting of the numerous professional objects which have attracted my attention since my residence in Paris, is this famous institution, a visit to which I enjoyed on the 5th of June, in company with Professor Mott, (who is intimately acquainted with Dr. Guérin, the chief director,) and Professor Ives, of Georgia. There is nothing equal, indeed, there is no institution similar to it in any other country; and nothing, in fine, is wanted, in order to render this establishment one of perfection for the noble purposes for which it was designed. The intelligence, genius, and affability of Dr. G., is admirably calculated to display, to the best advantage, to the numerous visiters professionally interested, the great advantages and benefits of the curious and new contrivances which he has brought to aid in the attainment of such desirable ends. Every species and degree of deformity, either accidental or connate, to which the human frame is liable, is here met with the appropriate means, moral, physical, and medicinal, to produce a permanent cure. A department of surgery so intricate and comparatively new, it may well be supposed, was not brought to such perfection without years of deep reflection, and numerous experimental essays on the part of its enlightened director; and it is equally gratifying and encouraging to the votaries of experimental philosophy to know, that in this instance, at least, laborious and well-directed effort has met with its merited reward—Dr. Guérin has secured to himself both fame and pecuniary compensation.

In the year 1830, the Royal Academy of Sciences published, as a prize subject, the following programme:—"To determine, by a series of facts and authentic observations, what are the advantages and disadvantages of the mechanical or gymnastic means applied to the cure of the deformities of the osseous system."

It was not until the year 1837 that Dr. Guérin considered the result of his labours sufficiently matured to enable him to present his memoir*, per

* The memoir was entitled "La Science des Difformités, placée, par la nature de ses faits, entre la physique the concours, for the grand surgical prize of ten thousand francs, which he obtained, by the unanimous report of the commissioners, MM. Dulong, Savart, Magendie, Serres, Larrey, Roux, and Double. During our visit, which was by appointment of Dr. Guérin, the numerous pupils from all parts of the civilized world, were variously employed in their gymnastic exercises, in a large and open hall devoted to the purpose, or were submitting to the various mechanical methods, as variously applied, for the correction of peculiar osseous deformities—the numerous kinds and grades of club-foot forming, though very numerous in themselves, but a small portion of the innumerable degrees of aberrations of the osseous frame, either congenital, or resulting from a vitiated physical education.

One large room was entirely devoted to the varied and curious machinery, for sitting, standing, and lying, in every position, so as to overcome, or counteract, every possible abnormal deviation of the spine or limbs. The gardens and forests attached to this institution, would well suit the luxurious retirement of a king, and, indeed, were once so appropriated. After a stroll along shaded gravel walks, and through a garden strewn with the richest flowers, Dr. G. inducted us into the *museum* of the establishment. Here are models in plaster or in wax, as well as the natural parts, collected from various sources, of the various cases of deformities treated by himself, and showing the changes produced in the different stages of the curative process. Of club-feet, there appeared to be no end to the variety. He removed the dressings from some cases under treatment, for our inspection. The tendo-achilles is divided by passing the needle under the skin, and over the tendon, cutting from without inwards, which obviates the possibility of endangering the integrity of the skin. Dr. G. politely invited us to assist at his next manual operations for the cure of club-feet, and kindly offered to notify us of the appointed day.

The ever-active surgeon of "La Charité," M. Velpeau, is now occupied in publishing a history of operative surgery. There is, undoubtedly, no other surgeon in Paris so well qualified for this delicate task—none so thoroughly acquainted with historical surgery, and, perhaps, in truth, so well disposed to do justice to the claims of others. He is almost the only lecturer who is in the habit of quoting American surgeons at all, and has requested several of our countrymen to prepare for his work an account of their own operations—one of whom, it is stated, asserts that he has performed the operation for lithotomy fifty times, forty successively terminating favourably, followed by ten cases successively fatal. It is to be hoped, for the credit of American surgeons, that such statements will be accompanied by such irrefragible proofs of their correctness, as to leave no room for future discreditable allegations and unnecessary cavil.

M. Velpeau appears to perform more operations in his hospital than any other surgeon, and is decidedly the favourite of the American students in Paris. His manners, though blunt and unpolished, and displaying none of the courtesies of life, are yet open, free, and familiar. Mr. Vanburen, of Philadelphia, who came out to Paris with me to pursue his professional studies for one year, gave M. Velpeau his early preference, and entered as "*interne*" several months since, still following the course of the hospital, and assisting in its duties, with unabated interest and devotion. One great advantage of such a course of studies is, that when he has completed them, and taken his degrees in the University of Pennsylvania, he will be enabled to enter his profession with the knowledge of a practitioner of several years' standing, in the ordinary course of things. M. V. has taken a great partiality for him, and always addresses him as "Monsieur le President," insisting on his being the son of the President of the United States, though this notion had been immediately corrected by Mr. Vanburen. This "sobriquet," given by M. Velpeau, has confirmed many of the medical class in the same notion of the high connections of Mr. Vanburen; and in one instance he has been the sufferer by it—his pocket-book having been recently abstracted by some impoverished son of Esculapius,—many of the kind swarm in Paris, from the provinces, wretchedly provided for, and who are put to all kinds of shifts and manœuvres, in order to make the two ends of the year meet. Some of them knowingly frequent *estaminets*, where horse-meat is eaten for beef-steaks, at a price one-half at which the "real sort" could be obtained raw in the markets—thus saving pence enough to purchase a new coat, and to partake of the indispensable delight of this genus, of dancing with their *cheres amies*, at the "Chaumiere," in the vicinity of Paris. One of these "gentlemen" carried his researches into Mr. Vanburen's pocket; he returned most of the contents of his et la Médecine, est destinée à nouer ces deux sciences à l' aide de la méthode expérimentale."

pocket-book the next day, with a polite note, stating that he (the thief) would be sorry to deprive the owner of his passports, tickets, certificates, &c., but that he must beg leave to reserve the pocket-book, as a memorial of so distinguished a personage as the "son of the President of the United States."

M. Velpeau's two last operations were interesting. He removed a very large calculus from the bladder of a female, from the provinces. He had previously notified the class of his intention to perform the high operation in this case; but, without any satisfactory reason for changing his mind, that I could see, he made the vesico-vaginal longitudinal section, taking care, however, to preserve the integrity of the urethra. The patient is doing well, ten days since the operation. He subsequently amputated the thigh of the shopkeeper noticed in a former letter, who had his thigh splintered and comminuted by a ball during the 12th May riot. M. V. could not at first convince the patient of the necessity of an immediate amputation, though he informed the class that he had scarcely any expectation of saving the limb—that most surgeons of experience have treated of the almost universal failure of the attempt to save the limb under similar circumstances. Accordingly, several weeks after the accident, the patient rapidly sinking under hectic fever, the operation was resorted to, cutting directly through the wound in the flesh, and amputating the bone some distance above the fracture. Not the least appearance of osseous reparation appearing at the fractured part, the patient now (several days after the operation) appears to be recovering, if he should escape the hospital fever which now prevails, together with erysipelas and gangrene, which too frequently end the operative process in these hospitals.

Early in June I received a polite note from M. Civiale to meet him at the Neckar hospital, to "assist" him in the operation for lithotritie. The patient was a middle-aged man from the provinces. It is really a most gratifying spectacle to witness the admirable dexterity with which this accomplished surgeon handles his instruments; I speak gravely when I assert that the feelings of this patient were rather pleasurable than painful, when under an operation, at one time considered the most frightful and dangerous of any to which a patient could be subjected. The man was conversing with a smile on his lips during the whole process; he was sounded, injected, and the instrument introduced, the stone crushed in its jaws, and literally eaten into a powder, in about five minutes, without the least delay, difficulty, or pain. The stone, it is true, from the appearance of the gravel, some of which was immediately voided, appeared to consist of urate of lime principally, but yet what a proud triumph of art over a horrible infirmity of our nature! No man can handle similar instruments with the dexterity of M. Civiale. I have never witnessed before a surgical operation with unmingled *pleasure*, principally because all suffering was absent. Some of the English surgeons have recently endeavoured to detract from M. Civiale's merits, asserting as an objection to this mode of operating, the comparatively greater liability to the return of the calculus. Admitting the truth of a position, not clearly made out, the superiority of the operation for the mechanical destruction of the calculus, over the usual murderous operation, in the hands at least of so skilful an operator as M. Civiale, is, indeed, incalculable.

I have visited the hospital "La Pitié," the theatre of the "good and evil deeds" of Lisfranc, much less frequently than the interest of the institution might justly claim, was it not three miles from my residence, and other institutions of equal claims being so much more convenient. So great has been the change in the personal appearance of M. Lisfranc during the last five years, since my last visit to Paris, that I was utterly unable to recognise him on our first interview. His gray and thinned locks, his bent form, and pale, haggard, and dissipated-looking countenance, have, in appearance, added twenty years to his life, and brought a constitution, originally the most robust and energetic, so much nearer the grave. His habits, irritability of temper, and violent passions, have had their share in inducing these results. His moral temperament is so decidedly méchant, that many of those (both compatriots and strangers) who most admire his professional talents, studiously avoid his social contact. With such developments added to his noted habit of abusing his contemporaries, it may well be supposed, he has secured himself few friends. Five years ago, I found, by repeated attention to his lectures, that M. Dupuytren was the hated object of his virulence and abuse. Among other opprobrious epithets applied at that time to his rival, I heard him say to his class that M. Dupuytren was "a damned old Hippopotamus," and repeatedly rated him for a thief and a liar; but now, that death has removed him from the sphere of his distorted vision, he has changed his notions. He constantly quotes him, (M. D.,) as the only surgeon worthy of being quoted, and he is the constant object of his adulation and praise. His vituperations have taken another direction, and poor Velpeau has to bear the brunt of his concentrated essence of rancour and animosity. In the delivery of his Leçons Orales, his manner is yet lively and energetic, boisterous and theatrical, his whole style much more suited to the noisy debates in the Chamber of Deputies, than to the dirty little lecture room where he daily holds forth. Some of our compatriots, who attend his daily rounds, prefer the practice of M. L. to that of any other surgeon, conceiving his treatment to be more philosophical, and regarding as of more consequence, constitutional remedies. The friends of M. L. attribute his soured and cynical temper to an unfortunate occurrence of some years' standing, viz., the loss of all his manuscript notes, which he had prepared with great labour for publication, and which were stolen one morning from his bureau. M. L. is reputed to have an extensive private practice.

Perhaps, some of your readers will suppose me very neglectful of the high claims of the celebrated Parisian practioners of medicine, from the slight notice, and unimportant space which their labours occupy in my communications; this is not because I know not how to estimate the value of their laborious pathological investigations, and the wonderful accurate prognosis to which they lead, but when it comes to the application of curative measures at the bed side—whether in the hospitals or in private life—my own experience forces me to pronounce these measures in a great degree efféte and nugatory.

We have far superior practitioners in the majority of medical men in our own country and in England.

On the 27th of May I accompanied my good old friend, Dr. Roberton,* to the house of the celebrated innovator in medicine, the venerable Dr. Hahnneman, who, for several years past has made Paris his home, and extensively practices his profession, chiefly receiving his patients at home, seldom visiting them at their houses. I was not long in observing that the old gentleman was not strictly Homœopathic in all his practices—he has a very pretty French wife, about thirty years of age, the Homœopathist being in his eighty-fifth year, as he himself informed me, adding "et cela est peu de chose," casting a side glance at the same time at his blooming wife who sat near; again, he smoked his pipe, on the Allopathic principle, it was scarcely out of his mouth the whole evening; and I am told that his charges to the faithful are truly enormous—he has lately received several hundred pounds sterling from an English nobleman for the mere attempt to cure a severe tic douloreux, by which he had been tormented for years; this was, indeed, "attacking the fortress of Gibralter with a pocket-pistol." In all other respects the soirèes of the venerable innovator are strictly Homœopathic.

He informed me that the Parisian Homœopathists were at present divided into two classes—the pure and the impure; the latter, prescribing on both principles, according to the whim of the patient; these, he said, he had discarded from his presence as false, as mere tradesmen, and as calumniators of the true science.

Dr. H. has considerable practice among the rich "*imaginaires*," both at home and abroad; he receives at home from 2 o'clock to three daily; he enjoys a fine establishment in a fashionable part of the city.

Dr. H. is small in stature; he has a fine Germanic skull, with a bald crown, and long white locks garnish his temples. His general resemblance was well represented by the late Charles Wilson Peale, of Philadelphia. He desired to know if I had his "Organon," and if I was acquainted with his disciple Dr. Herring, of Allentown, Pennsylvania; being answered in the affirmative, he expressed himself highly gratified with my acquaintance, requesting a frequent repetition of my visits, &c. He appeared desirous that his doctrines should extend to foreign countries—for he, at least, appears really to believe in them.

Monsieur Magendie's contemporaries, both at home and abroad, who occupy themselves in similar researches, urge him by their rivalry to constant operation; they complain of him that his numerous experiments on living animals in illustration of the nervous functions are not philosophically conducted, but that they are hap-hazard, and made without any definite object, and when, by chance, he falls on any novelty, or isolated fact, he makes the most of it, without referring with sufficient liberality to the prior discoveries of other labourers. Consequently, the

* Dr. Roberton is an eminent Scotch physician, who for the last forty years has been domiciliated in Paris, where, by his practice among the English, he soon secured a competence sufficient to enable him to retire from his profession, and has made his home an intellectual resource to the lovers of science of all nations.

numerous memoirs on this subject which he presents to the Institute, are followed by reclamations, from various quarters.

At a recent séance of the Academy of Sciences, he communicated the following facts, elicited by recent experiments. Alluding to the fact already ascertained, of the roots of the anterior spinal nerves receiving their sensibility from the roots of the posterior nerves, and that this acquired sensibility is derived from the circumference to the centre, he was desirous to ascertain if the same kind of influence existed in the fasciculi of the marrow itself. After having verified again that the posterior cords of the spinal marrow are endowed with exquisite sensibility, whilst the sensibility of the anterior cords is less pronounced, he divided, on one side, the posterior roots of a lumbar pair, making comparative observations at the same height, on the anterior fasciculus, and observed its sensibility to be very much diminished, if not altogether destroyed; this influence was probably transmitted by the motor roots, which remained untouched; to verify this, he left the sensitive roots in a state of integrity, and divided the motor roots; the same diminution of the sensibility of the cord at and above their origin was equally remarked.

From these experiments, M. M. infers, that, the posterior cord of the marrow—the sensitive roots—the ganglion—the rachidian nerve—the motor roots—and, in fine, the anterior or motor cord, form a sort of circular chain, each element of which serves to transmit sensibility from the posterior to the anterior cords.

M. Magendie continued to notice many facts in relation to the sensibility of the facial nerve, a sensibility which it acquires from the fifth pair, or sensitive nerve of the face. He remarked, that, of the three branches of the facial nerve, in the rabbit, the superior and inferior are entirely insensible, but that the middle branch offers, on the contrary, undoubted traces of sensibility. Astonished at this fact, M. M. frequently repeated the experiment, and always with similar results: but on the dissection of the head of a rabbit, this phenomenon was readily explained, by the discovery of a small filament of the fifth pair running to join the superior part of the middle branch; this filament being divided in a living rabbit, the middle branch of the facial nerve immediately lost all traces of sensibility, so long as this connection continued, the nerve was at the same time both sensitive and motor; as soon as this connection was dissolved, the isolated filaments preserved their proper characters of motor and sensible. The insensibility of the two branches of the facial nerve was not found to exist in the goat and the dog, and very probably does not exist in man. He called the attention of the class to a very interesting fact, viz.: the trunk of the facial nerve being divided, all the branches might, nevertheless, preserve their sensibility; the facial, although a motor nerve, might be the seat of neuralgia, and the section of its trunk with a view of a cure, might fail in producing this result.

The great *variety* of the intellectual objects of research discussed before the institute constitute one of the principal attractions of its weekly séances, and which renders these meetings of such general interest, that all the seats for spectators are frequently filled an hour before the séance is opened.

A thunder storm recently spent itself over Paris; the great dome of the "Invalids" was struck by lightning, occasioning considerable injury to the roof. A statement of the occurrence, with all the details, was immediately made to the institute by the governor of this magnificent institution; a commission was appointed to examine into all the circumstances, and M. Arago, the reporter, in a lucid statement which he made to the institute, among other remarks, affirmed that the injury resulted from a too sudden curve having been given to the lightning rod, when placing it on the dome, which circumstance he had already observed on other occasions deteriorates or destroys the conservative effects of the rod, inasmuch as the electric fluid is apt to continue its course in a straight line, when it arrives at this sudden curvature.

This same storm overtook the 58th regiment of the line, marching some miles distant from Paris, in one of the departments. Fifty of the soldiers were struck down by the lightning; they discharged blood from their ears, nose, and mouth, but all recovered except two; it is not stated whether or not they were marching with fixed bayonets at the time.

M. Amussat has communicated to the institute an account of a surgical operation which he has recently performed, and which from its novelty attracted some attention, although some conclude that the morbid state resulting from the operation is worse than that which it was intended to remedy; he produced an artificial anus by opening the colon, without including in the incision the *peritoneal* coat of the intestine; the

*peritoneal sac* preserving its *integrity*. The patient was a respectable female, in whom a large abdominal tumor had, by pressure on the colon, entirely obstructed the passage, resulting in an enormous distention of the intestine by fecal matter; a tranverse incision was made in the right lumbar region near the spine down to the tumour, being that portion of the ascending colon nearest the spine, and where the lamina of the mesocolon was separated by the distention of its accumulated contents. The patient, now some weeks since the operation, is represented as in a fair way, the more pressing symptoms being relieved.

M. Daguerre has at length reaped some pecuniary reward from the Government for his photogenic discoveries; the Chamber of Deputies having voted him an annuity of $1200, the half of which will be continued to his family if his widow survives him. The son of the late M. Niepse, who was originally associated with M. D., in these discoveries, is to receive $600 per annum. M. D.'s secret will, of consequence, be soon made public. Portraits are already taken by this method at about $3 each, on prepared paper or metallic plates.

The science of phrenology has not so many votaries in Paris at present as during the days of Gall and Spurzheim; there is, however, a very respectable society here, chiefly composed of physicians. I visited their rooms, lately, in the Rue de Seine, St. Germain, in company with Dr. Combe, of Edinburgh, and Dr. Robertson, the last named presided. The cabinet of the society consists of an extensive and valuable collection, principally of plaster casts.

Dr. Voisin, one of the most zealous members, has, in unison with Dr. Falret, carried out the principles of this science, in their application to the cure of mental alienation, on a grand scale; their hospital at Vanvres, near Paris, is an admirable institution in all respects; I passed the greater part of a day there, and was received with great respect by these enterprising and intelligent directors, or rather proprietors: from their published reports, their success in the treatment of their patients, would seem to be paramount to the importance of the object; the inmates appeared to me to be more comfortable and happy than those of any similar institution that I have visited.

---

The Coroner of the Western Division, Middlesex, London, says there are held annually, 1500 inquests—900 of which are caused by intoxicating drinks.

